# Supplementary material for: Assessing perceptions of establishing a vaccine pooled procurement mechanism for the Western Pacific Region
Source: PLOS Glob Public Health. 2022 Aug 12;2(8):e0000801. doi: 10.1371/journal.pgph.0000801 (PMC10021624; doi:10.1371/journal.pgph.0000801)
Supplement: S1 Table — (PDF) [file pgph.0000801.s001.pdf]

S1 Table. Comparison of themes within vaccine pooled procurement surveys[1]

**Deroeck question guide, 2003**[2] (Number of countries=4)	**WHO/EURO rapid survey, 2003**[2] (Number of counterparts surveyed=15)	**WPRO survey, 2019**[3] (Number of countries =13)
Current situation<br>Basic vaccine information	Introductory questions	Country background
Vaccine procurement procedures and system	Procurement assessment tool<br>Procurement process<br>Organization<br>Legal basis and infrastructure<br>Budgeting and finance	Country-specific procurement process<br>Vaccine pricing
Quality assurance	Ensuring quality<br>Delivery distribution and cold chain	Vaccine delivery and quality
Feasibility of group tendering or procurement<br>Options for group tendering or procurement system	---	Perceptions on developing a pooled vaccine procurement mechanism
Questions for financial people in ministry of health<br>Questions for procurement office<br>Questions for NRA officials<br>Questions for senior ministry of health officials<br>Questions for finance and trade/economic affairs ministry officials	---	---

[1] Themes are not in the same order as they appearance in the surveys
[2] Deroeck et al 2003
[3] Survey assessing perceptions of establishing a vaccine pooled procurement mechanism for Western Pacific Region
