# Supplementary material for: Assessing perceptions of establishing a vaccine pooled procurement mechanism for the Western Pacific Region
Source: PLOS Glob Public Health. 2022 Aug 12;2(8):e0000801. doi: 10.1371/journal.pgph.0000801 (PMC10021624; doi:10.1371/journal.pgph.0000801)
Supplement: S2 Table — (PDF) [file pgph.0000801.s002.pdf]

S2 Table. Summary of respondents to survey assessing perceptions of establishing a vaccine pooled procurement mechanism for Western Pacific Region

**Stratification of survey respondents** (Denominator)	**N**	**(%)**
**Countries**[1] (N=27)	13	48%
**Country income groups**[2]		
High-income countries	6	46%
Upper middle-income countries	3	23%
Lower middle-income countries	4	31%
**Eligible to be surveyed**[3] (N=80)	17	21%
Ministry of Health (N=76)	15	20%
NITAG members (N=3)	2	67%
Ministry of finance (N=1)	0	0%
**Respondents** (N=17)		
Immunization managers	6	35%
NITAG members	2	12%
Other[4]	9	53%
Currently in office	15	94%
**Years of experience**		
<1 year	1	6%
1-3 years	2	12%
3-5 years	2	12%
5+ years	12	71%

NITAG – National Immunization Technical Advisory Group

[1] Country list includes Australia, Brunei Darussalam, Cambodia, China, Cook Islands, Fiji, Japan, Kiribati, Lao People's Democratic Republic, Malaysia, Marshall Islands, Micronesia, Mongolia, Nauru, New Zealand, Niue, Palau, Papua New Guinea, Philippines, Republic of Korea, Samoa, Singapore, Solomon Islands, Tonga, Tuvalu, Vanuatu, Vietnam out of which Australia, Brunei Darussalam, Cook Islands, Malaysia, Mongolia, New Zealand, Niue, Palau, Papua New Guinea, Philippines, Samoa, Tuvalu, Vietnam had national counterparts that provided a response to the survey (areas were not included in the survey)

[2] Based on 2016 World Bank income classification; all countries represented had at least one respondent from the national ministry of health

[3] 80 persons were formally invited to participate to complete the online survey

[4] Answer includes vaccine procurement-related positions (2), Immunization/Expanded Program on Immunization team member (5), Surveillance (1), Registrar (1)
