# Supplementary material for: Assessing perceptions of establishing a vaccine pooled procurement mechanism for the Western Pacific Region
Source: PLOS Glob Public Health. 2022 Aug 12;2(8):e0000801. doi: 10.1371/journal.pgph.0000801 (PMC10021624; doi:10.1371/journal.pgph.0000801)
Supplement: S3 Table — (PDF) [file pgph.0000801.s003.pdf]

S3 Table. Summary of vaccine prices and procurement mechanisms as reported in survey assessing perceptions of establishing a vaccine pooled procurement mechanism for Western Pacific Region

Vaccines	Included in NIP (N=13)	Vaccine prices (US$)			Procurement mechanism					
	n[1]	Avg.	n[2]	Range[3]	Gavi through UNICEF /SD	Gavi through independent supplier	UNICEF /SD (non-Gavi)	International vaccine manufacturer	National vaccine manufacturer	Other
Bacillus Calmette–Guérin vaccine (BCG)	11	$2.36	5	$0.16 - 8.75	-	-	4	3	1	-
Hepatitis B (Birth Dose) [4]	13	$7.72	6	$0.20 - 20.88	-	-	3	4	1	1
Diphtheria-Tetanus-Pertussis containing vaccine (DTP-CV)[5]	13	$15.72	7	$0.15 - 60.00	1	-	3	5	-	2
Haemophilus influenzae type B (Hib) Vaccine	5	$9.27	3	$10.00 - 14.31	-	-		3	-	2
Measles containing vaccine[6]	13	$7.98	6	$1.20 - 22.00	-	-	4	5	1	2
Inactivates polio vaccine	7	$3.07	2	$1.90 - 4.24	1	-	2	-	-	1
Oral polio vaccine	6	$0.15	2	$0.10 - 0.20	-	-	2	-	1	1
Pneumococcal conjugate vaccine[7]	6	$48.93	3	$3.30 - 140.00	-	-	1	2	-	2
Rotavirus vaccine[8]	4	$36.75	2	$3.50 - 70.00	-	-	-	1	-	1
Human Papillomavirus vaccine[9]	8	$51.00	4	$7.72 - 180.00	-	-	1	4	-	2
Japanese Encephalitis vaccine	3	$12.00	1	-	-	-	-	1	1	-
Rubella vaccine	1	-	-	-	-	-	-	-	1	-
Paediatric influenza vaccine	1	-	-	-	-	-	-	1	-	-
Current payment regulations and processes for each supplier		Upon receipt of goods			-	-	2	5	3	2
		Before receipt of goods			1	-	2	1	-	1
		Not Applicable			2	4	2	1	3	3
		Unknown			4	4	4	3	2	3

1 Number of respondents that reported having the vaccine universally offered within their National Immunization Program (NIP)
2 Number of respondents that provided an answer for the survey question
3 Lower and upper-bound vaccine prices as reported by surveyed national counterparts
4 Birth dose of the Hepatitis B vaccine
5 Diphtheria, tetanus, pertussis containing vaccine presentations within routine vaccine schedule specified by 13 country-respondents as following: DTaP-Hib-HepB-IPV (4), DTwP-Hib-HepB (5) DTaP-Hib-IPV (1), DTaP-HepB-IPV (1)
6 Presentations specified by 11 country-respondents as the following: Monovalent MCV, Measles and Rubella vaccine - MR (1), Measles, Mumps and Rubella vaccine - MMR (9)
7 Three countries reported using PCV10 and four countries reported using PCV13
8 Represents four countries reported using Rotarix (2-dose series) and one country using RotaTeq (3-dose series)
9 Represents three countries reported using Ceravix and four countries reported using Gardasil-9
