# Supplementary material for: Assessing perceptions of establishing a vaccine pooled procurement mechanism for the Western Pacific Region
Source: PLOS Glob Public Health. 2022 Aug 12;2(8):e0000801. doi: 10.1371/journal.pgph.0000801 (PMC10021624; doi:10.1371/journal.pgph.0000801)
Supplement: Attachment — Submitted filename: WPRO_Response_Letter_20220614.docx [file pgph.0000801.s005.docx]

14 June 2022

Dear Dr. Flaxman,

**Manuscript “*Assessing perceptions of establishing a vaccine pooled procurement mechanism for the Western Pacific Region*”**

Thank you for your feedback on our manuscript and for kindly considering its publication in PLOS Global Public Health.

Please see below our responses to the Editor and Reviewer comments and our suggested edits:

**Academic Editor’s (AE) Comments**

**Comment-1:** Country-specific procurement process (Line 149 - 191) - it could be a bit overwhelming to have all of these results together in a single paragraph. Consider restructuring thematically, into smaller paragraphs, grouping results from related questions together.

***Authors’ Response-1:*** *Section listed, “Country-specific procurement process”, i.e. lines 149-191*

*has been broken down into five paragraphs that have received the following titles: (1) Vaccine forecasting requirements and national approval timelines, (2) National tender and bidding processes, (3) Vaccine Specifications, Licensing & National Regulatory Authorities, (4) Implementation of National Immunization Technical Advisory Group Recommendations, (5) Vaccine Supply and Stock-Out Frequency. We hope that this theme specific paragraph division will provide additional clarity to the section and results.*

**Comment-2:** Same comment for Vaccine Prices and Procurement Mechanisms Section (Line 196 - 239)

***Authors’ Response-2:*** *Section listed, “Vaccine Prices and Procurement Mechanisms”, i.e. lines 196-239 has been broken down into four paragraphs that have received the following titles: (1) Perception on Vaccine Price Fairness and Affordability, (2) Knowledge on Vaccine Pricing Resource and Reporting on Vaccine Pricing, (3) Vaccine Price Reporting by Income Classification, (4) Vaccine Procurement Mechanism Used.*

**Comment-3:** Supplementary Table 3, and in particular, the range in vaccine prices that you found seem worthy of more prominent reporting, perhaps even in a figure.  Am I reading correctly that the reported Hep B vaccine price varies 10,000%, from $0.20 to $20.88?  That is just striking.

***Author’s Response-3:*** *Due to the limited number of countries reporting vaccine pricing, along with the inconsistent data reported across vaccines, a decision was made to avoid drawing too many conclusions regarding the vaccine pricing data reported.*

**Reviewer's Comments:**

**Comment-1:** The table 1 is clear and yet not very easy to grasp at a glance. I would suggest that the numbers in the three columns be replaced by a horizontal stacked bar-chart (with numbers printed in the bars), which would make it immediately clear which characteristics are generally found in large numbers of countries – especially if colour codes are used. I would then present the bar-sections as yes (green) undecided (yellow) and no (red).

***Author’s Response-1:*** *Edits have been made accordingly to Tables 1 (pg. 11) and 2 (pg. 16) to make the results more visually intuitive and clear.*

**Comment-2:** Table 3: I would re-order them, starting always with the statement of highest agreement (e.g. timely vaccine delivery, 10 points)

***Author’s Response-2:*** *Edits have been made accordingly to display the* *responses of Table 3 (pg. 18) in descending order.*
